# DEmiRNA-mRNA regulatory network reveals miR‐122-5p as a regulatory factor of arginine metabolism in necrotizing enterocolitis

**DOI:** 10.3389/fgene.2024.1480431

**Published:** 2025-01-22

**Authors:** Zhili Ding, Ting Guo, Qiang Tang, Yaqiang Hong, Zhibao Lv, Li Lu, Wenjun Zhuang

**Affiliations:** ^1^ Department of General Surgery, Affiliated Changzhou Children’s Hospital of Nantong University, Changzhou, Jiangsu, China; ^2^ Department of General Surgery, Shanghai Children’s Hospital, School of Medicine, Shanghai Jiao Tong University, Shanghai, China

**Keywords:** neonatal necrotizing enterocolitis, miR-122-5p, arginine metabolism, intestinal stem cell, biomarker

## Abstract

**Objective:**

Necrotizing enterocolitis (NEC) is a gastrointestinal emergency with relatively high morbidity and mortality in neonates. The role of microRNAs (miRNAs) in NEC is not yet entirely clear. This study aimed to explore the mechanism of miR-122-5p in NEC.

**Methods:**

Differentially expressed (DE) miRNAs were sequenced in control and NEC mice. The DEmiRNA-mRNA regulatory network was constructed and the bioinformatics analysis was performed to identify the target mRNAs and potential roles of the DEmiRNAs. The miR-122-5p activation was explored *in vitro* in the human intestinal epithelial cell (FHs74Int) and rat intestinal epithelial cell (IEC-6). *In vivo*, mice were transinfected with miR-122-5p inhibitor before the NEC occurred. Mass spectrometry was used to qualify the concentrations of amino acids, and the viability of intestinal stem cell (ISC) was accessed to verify the biological function.

**Results:**

Preliminarily, 15 miRNAs were found to be differentially expressed between NEC group and control group. Subsequent bioinformatics analysis revealed that miR-122-5p significantly contributes to the arginine metabolism in NEC through the DEmiRNA-mRNA regulatory network, with PRODH2 and ALDH18A1 being identified as its target genes. *In vitro*, miR-122-5p mimic inhibited the expression of PRODH2 and ALDH18A1 in the FHs74Int cells and IEC-6 cells*. In vivo*, inhibition of miR-122-5p led to increased expression of PRODH2 and ALDH18A1, along with elevated arginine levels. Following transfection with a miR-122-5p inhibiting adenovirus, the survival rate of NEC mice improved, and intestinal injury was alleviated.

**Conclusion:**

MiR-122-5p inhibition could impact arginine metabolism by targeting PRODH2 and ALDH18A1, thereby mitigating intestinal injury in NEC.

## Introduction

Necrotizing enterocolitis (NEC) is a common neonatal gastrointestinal emergency, especially in premature infants, with a morbidity rate of 6%–10% and a high mortality rate of up to 20%–30% (Cai et al., 2022a; Bethell and Hall, 2023). NEC is believed to result from the interaction of multiple factors, including prematurity, formula feeding, dysbacteriosis, ischemia, and hypoxia, leading to subsequent intestinal barrier damage (Robinson et al., 2018; Frost et al., 2017; Coggins et al., 2015). Despite extensive research, the etiopathogenesis of NEC remains unclear (Neu et al., 2018; Mani et al., 2023).

Nutritional supplementation, such as L-arginine, may alleviate tissue damage from intestinal ischemia and promote intestinal mucosal healing in premature infants (Neu, 2007; Shah et al., 2017; Bober-Olesińska and Kornacka, 2005). Arginine is an essential amino acid synthesized from citrulline and proline primarily in small intestine, and it is involved in several physiological and metabolic pathways (Jessica et al., 2020; Garg, 2018). L-arginine can be converted to endogenous nitric oxide (NO) by endothelial nitric oxide synthase (eNOS), thereby enhancing intestinal perfusion, reducing the detrimental effect of endothelial receptor toll-like receptor 4 (TLR4) activation and mitigating NEC intestinal damage (Yazji et al., 2013). Moreover, L-arginine plays a crucial role for intestinal stem cells (ISCs) proliferation and intestinal epithelial renewal through the Wnt/β-catenin signaling pathway in mice, small intestinal (SI) organoids, and ISC-Paneth cell co-cultured models (Hou QH. et al., 2020). Although arginine is beneficial for gastrointestinal development, few reports have focused on the pathogenesis of arginine metabolism in NEC.

MicroRNA (miRNA) is a short non-coding RNA molecule that decreased the target messenger RNAs (mRNAs) levels through post transcriptional regulation (Chen et al., 2019). Over the past several years, studies have elucidated specific mechanisms of miRNAs in NEC, including intestinal barrier rehabilitation, dysregulation of apoptosis and inflammatory infiltration (Cai et al., 2022b; Wang et al., 2012). MiR-124 has been found to be upregulated in NEC, promoting the intestine cell apoptosis and inflammatory infiltration via inhibiting Toll-like receptor 9 (TLR9) expression by targeting rho-associated coiled-coil-containing protein kinase 1 (ROCK1) (Yin et al., 2019). For inflammatory infiltration response, miR-146a-5p can attenuate inflammation and intestinal damage in the NEC by inhibiting leucine-rich repeat-containing protein 3 (NLRP3) inflammasome downstream inflammatory factors and chloride intracellular channel protein 4 (CLIC4) membrane expression (Chen et al., 2021). Moreover, network analysis has suggested a potential association of miR-146-3p and miR-451 with arginine metabolism in NEC and spontaneous intestinal perforation (SIP) tissues (Ng et al., 2015). However, the mechanism underlying the correlation between miRNAs and arginine metabolism in NEC remains unknown yet.

In this study, we explore the expression profiles of miRNAs in NEC mice compared with the control group, investigating the potential functions of these DEmiRNAs through the construction of a DEmiRNA-mRNA regulatory network. Additionally, the IEC‐6 cell line and the NEC mice model were used to verify the potential pathogenesis of NEC. Our findings might provide new insights into the mechanism of NEC.

## Methods

### MiRNA and mRNA sequencing

Six miRNA libraries were constructed from the control group and the NEC group. Sequencing was performed by Shanghai OE Biotechnology Co., Ltd. (China). Total RNA was extracted using Trizol reagent (Invitrogen, Carlsbad, CA) and chloroform, and RNA integrity was assessed using the RNA Nano 6000 Assay Kit of the Agilent Bioanalyzer 2100 system (Agilent Technologies, CA, United States). The mRNA sequencing libraries were generated using NEBNext^®^ UltraTM RNA Library Prep Kit for Illumina^®^ (NEB, United States) following manufacturer’s recommendations, with index codes added to attribute sequences to each sample. The clustering of the index-coded samples was performed on a cBot Cluster Generation System using TruSeq PE Cluster Kit v3-cBot-HS (Illumia) according to the manufacturer’s instructions. After cluster generation, the library preparations were sequenced on an Illumina Novaseq platform and 150 bp paired-end reads were generated.

The miRNA sequencing libraries were generated using NEBNext^®^ Multiplex Small RNA Library Prep Set for Illumina^®^ (NEB, United States) following manufacturer’s recommendations and index codes were added to attribute sequences to each sample. The clustering of the index-coded samples was performed on a cBot Cluster Generation System using HiSeq Rapid Duo cBot Sample Loading Kit (Illumia) according to the manufacturer’s instructions. After cluster generation, the library preparations were sequenced on an Illumina Hiseq 2500 platform and 50bp single-end reads were generated.

### Target prediction for the DEmiRNAS

To predict target mRNAs of the DE miRNAs, R DESeq2 (version 1.34.0) package was performed. Bonferroni correction or Benjamini–Hochberg False Discovery Rate (FDR) correction were also applied to control for multiple comparisons in the analysis of DE miRNAs. However, no statistically significant results were observed after Bonferroni or FDR correction (Supplementary Table S1). We also evaluated the DEmRNAs with FDR correction (Supplementary Table S2). Finally, the significant DEmRNAs between NEC and control samples were identified with *p*-value < 0.05 and |log2 (Fold Change) | > 1.0. To assess the potential functions of the DE genes, Gene Ontology (GO) and Kyoto Encyclopedia of Genes and Genomes (KEGG) pathway enrichment analyses were performed using DAVID database (https://david.ncifcrf.gov/). The GO analysis included three categories: biological process (BP), cellular component (CC) and molecular function (MF).

### Cell culture and treatment

The FHs74Int cells and IEC-6 cells were obtained from the American Type Culture Collection (ATCC), cultured in DMEM supplemented with 10% fetal bovine serum and 1% penicillin/streptomycin, in the humidified atmosphere of 37°C and 5% CO2. For the next experiment, medium was replaced by Opti-DMEM, the FHs74Int cells and IEC-6 cells were pre-incubated with miR-122-5p overexpression sequence. After 48h, the treated cells were collected for subsequent analysis.

### Mice and treatment

C57BL/6J mice (1 day old) were obtained from Shanghai Jiesijie Laboratory Animal Co., Ltd. At day 5, the mice were randomly assigned to two groups. The control group received maternal milk without hypoxia and cold stimulation. NEC group was induced using formula gavage every 4 hours using Esbilac formula (Pet-Ag, Abbott Laboratories) and exposed to hypoxia treatments (99.9% N2 for 100s) and cold (4°C for 10min) twice daily in a hypoxic chamber for 4 days. For subsequent biological function validation, the pups were divided into four groups totally for adenovirus intraperitoneal injection: the scrambled mice (scrambled), the scrambled mice injected with inhibiting adenovirus (miR-22-5p-inhibitor + scrambled), the NEC mice (NEC), the NEC mice injected with inhibiting adenovirus (miR-22-5p-inhibitor + NEC). Daily body weight and survival times of pups were recorded. Euthanasia was conducted at 96h, then the ileum was collected and divided into two parts: one stored in 10% formalin for Hematoxylin-Eosin Staining and immunofluorescence, and the other stored at −80°C for Quantitative Real-Time PCR, and RNA-sequencing. All the animal experiments were approved by the Animal Care Committee of the Children’s Hospital of Shanghai.

### Mass spectrometry

The amino acid concentrations in frozen tissues were measured by mass spectrometry based on our previous study (Guo et al., 2023). Deuterium-labeled internal standards, standard products, quality control products, and reagents were purchased from Guangzhou Clin Meta Medical Device Co., Ltd. First, 20 μL of serum (standard products/quality control products) and 80 μL of dilution solution were vortexed for 5 min, 10 μL of diluted serum and 100 μL of ice-cold methanol (contain partial internal standards) were vortexed for 5 min. Then, 50 μL aliquots of the supernatant were obtained into autosampler vials by centrifugation at 13,000 rpm for 5 min, with nitrogen until completely dry at 50°C. Next, the samples were vortexed with 500 μL of freshly prepared derivatization reagents for 30 min, with nitrogen until completely dry at 60°C.Then, 100 μL diluted aliquots were added to vials and vortexed for 5 min. Take 2 μL sample mixed well injecting onto a separation column (ACE EXCEL 3 AQ, 100 × 3.0 MM), analysis was then performed using LC–MS/MS (Shimadzu LCMS-8040CL) to measure the concentrations of all amino acids.

### Quantitative real-time PCR

Total RNA was extracted from the treated IEC-6 cells and experimental intestinal tissues of mice using Trizol following the manufacturer’s instructions. To measure the miRNAs expression levels, RNAs were transcribed by stem-loop RT primer using a reverse transcription kit (Sangon Biotech Co., Ltd.). For other target genes, the cDNAs were reversed using a Takara reverse transcription kit (AG, Hunan, China). The microRNA qPCR kit (Sangon Biotech Co., Ltd.) and quantitative mRNA kit (Yeasen, Shanghai, China) were used for microRNA and mRNA quantitative real-time PCR analysis respectively to measure the expression level in cells and tissues according to the manufacturer’s instructions. U6 (for the miRNAs) and β-Actin (for the mRNAs) were used as the internal reference. The primer sequences involved were listed in the Supplementary Table S3.

### Hematoxylin-eosin (H&E) staining

Intestine samples taken out from 4% formalin were dehydrated with a graded series of ethanol, cleared in xylene, and embedded in paraffin. Hematoxylin and eosin (H&E) staining was performed on 6 μm thick sections sliced using microtome separately. After H&E staining, a histopathological examination was observed under an optical microscope.

The NEC scoring system was as follows: Score 0: normal, Score1: minor swelling and separation on submucosal or lamina propria, score 2: moderate separation on submucosal or lamina propria, submucosal or muscular edema, score 3: severe submucosal or lamina propria separation, muscular edema, partial loss of the villus, score 4: loss of intestinal villus with intestinal necrosis. A score equal to or >2 suggests the occurrence of NEC.

### Immunofluorescence staining

Paraffin sections were soaked in xylene and serially passed through decreasing concentrations of ethanol, then antigen retrieval was conducted. Sections were washed three times with TPBS and blocked with 5% bovine serum albumin for 60 min at room temperature. Subsequently, sections were incubated overnight at 4°C with primary antibodies, anti-Ki67 (Servicebio, Wuhan, China) or Olfm4 (Cell Signaling Technology, Danvers, MA) followed by incubation with horseradish peroxidase-coupled secondary antibodies at room temperature for 1 h. Finally, DAPI solution was used for staining, images were captured under a fluorescence microscope.

### Statistical analysis

Statistical calculations were carried out using SPSS 19.0. Student’s t*-*test or the Mann-Whitney test was conducted according to the normality of data distribution to compare the two groups. The log-rank test was used for survival analysis. Statistically significant was defined as *p* < 0.05 (∗), *p* < 0.01 (∗∗), and *p* < 0.001 (∗∗∗).

## Results

### Identification of differentially expressed miRNAs in NEC

**FIGURE 1:**
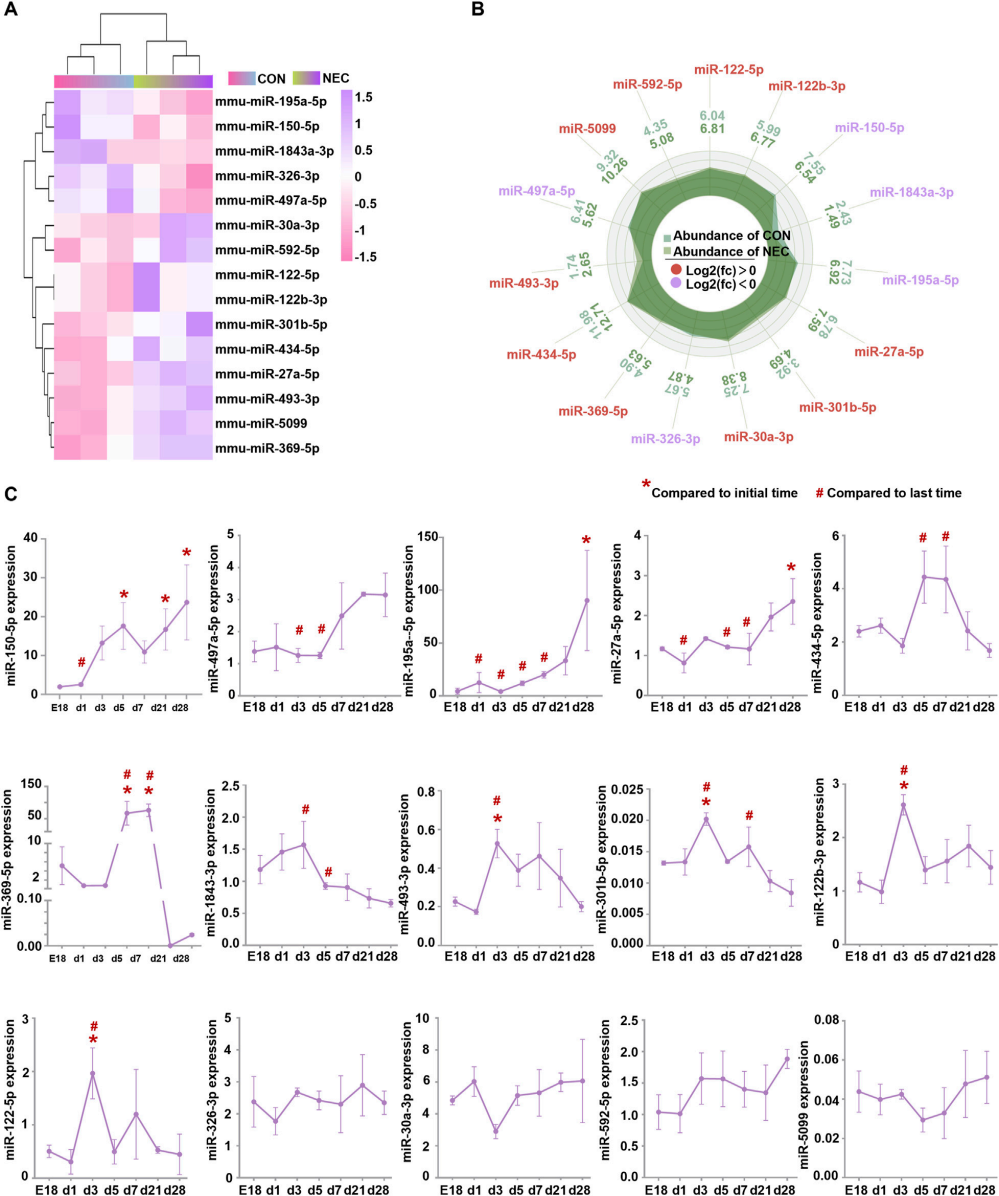
DEmiRNAs are involved in intestinal development. **(A)** Heatmap of fifteen DEmiRNAs (|log2 (Fold Change) | > 1.2, *p* < 0.05) between NEC and control groups. The color in heatmap reflects the degree of expression. High expression is showed in purple and low expression is showed in pink. **(B)** Histogram of DEmiRNAs. High and low expression of genes respectively based on the cut-off values of log2 (Fold Change). **(C)** The levels of DEmiRNAs were measured by quantitative RT-PCR at E18, day 1, day 3, day 5, day 7, day 21 and day 28. Results were expressed as mean ± SEM. Initial time: mice at embryonic day 18 (E18); Last time: postnatal day28.

To explore the role of miRNAs in NEC, we conducted miRNA high-throughput sequencing and identified a total of 604 miRNAs (Supplementary Table S1). 15 differentially expressed miRNAs (DEmiRNAs) were found, including five downregulated miRNAs and 10 upregulated miRNAs (Figures 1A, B).

We analyzed the expression pattern of these DEmiRNAs during the intestinal development process. Ileum were harvested from C57BL/6J mice at embryonic day 18 (E18) and postnatal days (d)1, 3, 5, 7, 21 and 28. PCR results revealed that 11 miRNAs changed during the intestine development, excepting miR-326-3p, miR-30a-3p, miR-592-5p, miR-5099 (Figure 1C). Additionally, we found a significant increase in the expression levels of miR-150-5p, miR-497a-5p, and miR-195a-5p from birth to day 28. However, their expression levels were downregulated in our NEC tissues by high-throughput sequencing analysis. MiR-434-5p, miR-369-5p, miR-493-3p, miR-301b-5p, miR-122b-3p, and miR-122-5p showed a significant increase in expression levels from birth to about day 3 or day 5 but subsequently decreased back to embryologic levels, nevertheless their expression levels were upregulated in our NEC tissues by high-throughput sequencing analysis. No significant changes were observed in miR-326-3p, miR-30a-3p, miR-592-5p, and miR-5099. However, their expression levels were dysregulated in the NEC tissues by high-throughput sequencing analysis (Table 1). These findings suggested that most of the DEmiRNAs changed and maybe involved in the maturation process of intestine and the progress of NEC.

**TABLE 1:** The expression level of DEmiRNAs in the intestinal maturation process and the progress of NEC.

MiRNA name	Expression level	
	In the intestinal maturation process	In the NEC progress
miRNA-150-5p, miR-497a-5p, and miR-195a-5p	↑	↓
miR-434-5p, miR-369-5p, miR-493-3p, miR-301b-5p, miR-122b-3p, and miR-122-5p	↓^*^	↑
miR-326-3p	→	↓
miR-30a-3p, miR-592-5p, and miR-5099	→	↑
miR-27a-5p	↑	↑
miR-1843-3p	↓	↓

↑: significantly upregulated; ↓: significantly downregulated.

→: no significant changes; *: increased initially to d3/d5, followed by a decrease.

### Prediction of DEmiRNA targets and construction of DemiRNA-mRNA regulatory network

**FIGURE 2:**
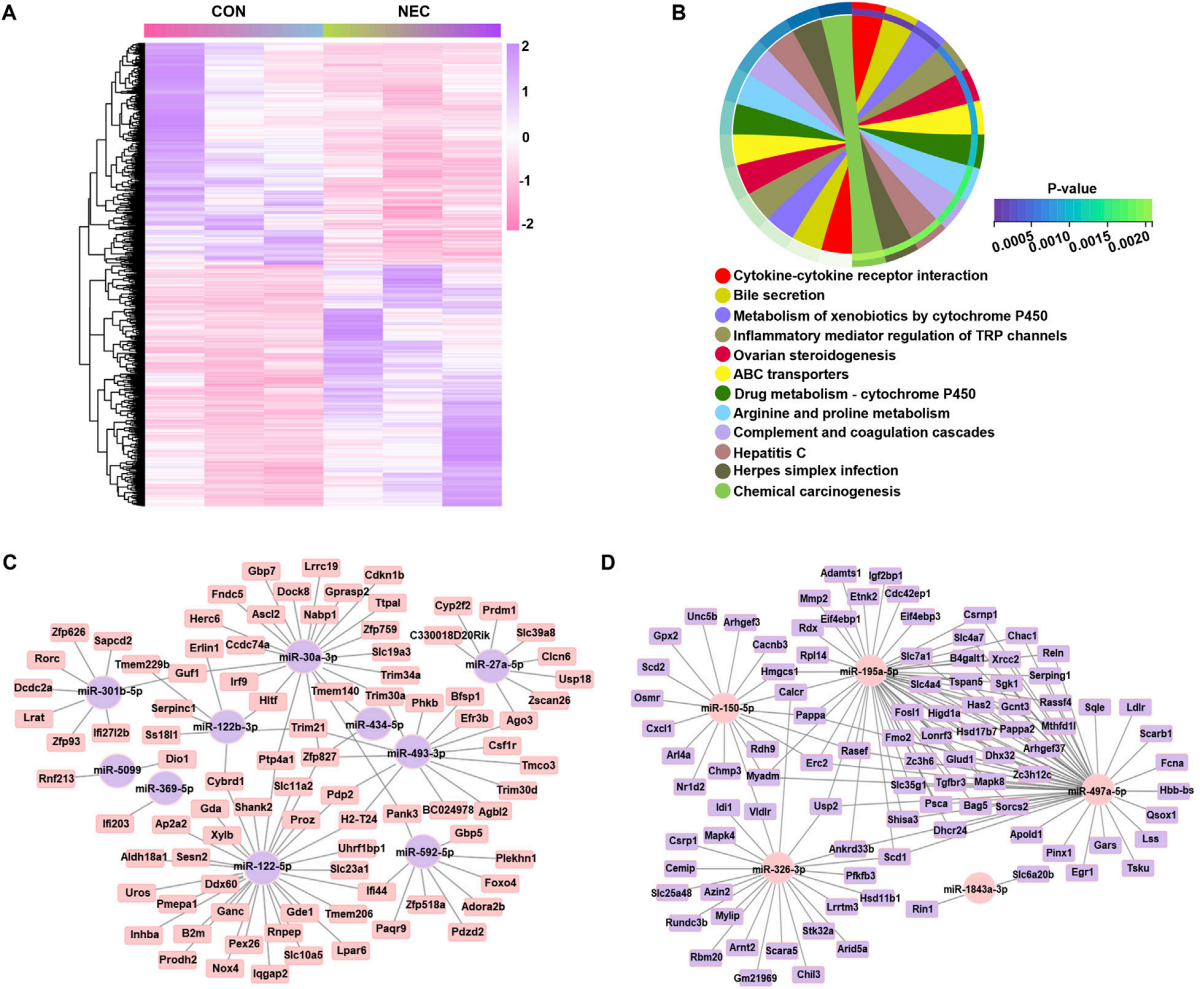
Gene co-expression network regulated by DEmiRNAs. **(A)** Heatmap of DEmRNAs (|log2 (Fold Change) | > 1, *p* < 0.05) in two groups, with each line representing a DEmRNA. High levels are shown in purple; low levels are shown in pink. **(B)** KEGG pathways significantly enriched with altered genes, as analyzed with the DAVID tool (dashed line: *p*-value equal to 0.05). **(C)** The regulatory network of upregulated miRNAs. **(D)** The regulatory network of downregulated miRNAs. Elliptical nodes represent miRNA; Square nodes represent mRNA; Purple represents upregulated miRNA or mRNA; Pink represents downregulated miRNA or mRNA; Black lines represent connections between mRNA and miRNA. The network was visualized using Cytoscape version 3.6.0 software.

To predict the function of DEmiRNAs, we constructed the regulatory network combined with the expression prolife of mRNA. We screened all identified genes by applying the certain criteria (*p*_adj_ < 0.05) and finally identified a total of 1212 DEmRNAs (Figure 2A). Considering the potential roles of these 1212 DEmRNAs, we performed the KEGG, which showed significant enrichment in 12 pathways, primarily associated with immune response (cytokine-cytokine receptor interaction and inflammatory mediator regulation of TRP channels) as well as metabolism pathway (bile secretion, metabolism of cytochrome P450 and amino acid metabolism) (Figure 2B). The putative targeted DEmRNAs of DEmiRNAs were predicted using four bioinformatic algorithms (TargetScan 8.0, miRDB, DIANA-TarBase and miRTarBase) where a gene was considered a candidate if it was predicted in at least one database. Since miRNAs typically downregulate their target mRNA expression, we further narrowed down the candidate range by selecting genes reversely expressed with miRNA. Removing the duplicated mRNAs, a miRNA-mRNA network was formed by 10 upregulated miRNAs and 89 downregulated mRNAs, and another miRNA-mRNA network was formed by five downregulated miRNAs and 96 upregulated mRNAs (Figures 2C, D). Consequently, the comprehensive analysis led to the identification of 185 target genes which potentially regulated by the DEmiRNAs.

### Functional analysis of DEmiRNAs and target mRNAs

**FIGURE 3:**
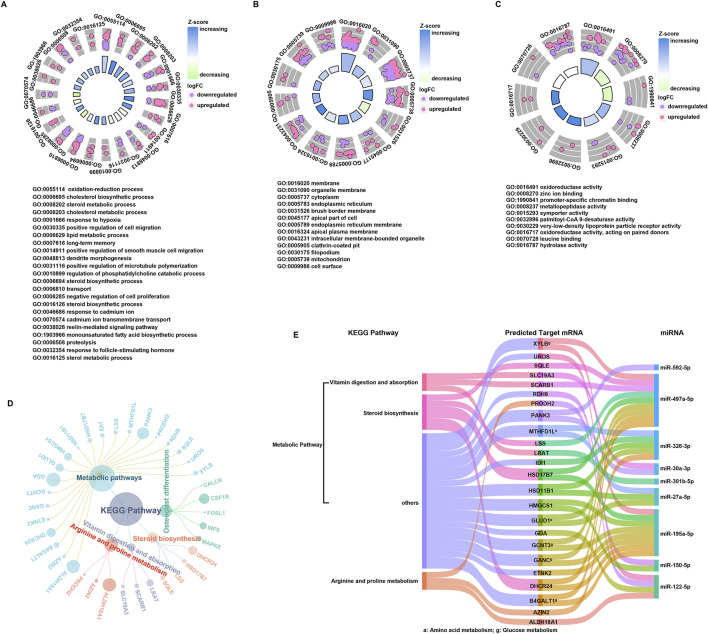
Functional annotation of DEmiRNAs and targeted mRNAs. **(A–C)** Histogram of GO classification for targeted mRNAs. The results were summarized into three main categories: biological process **(A)**, cellular component **(B)** and molecular function **(C)**. **(D)** KEGG pathways enriched in targeted mRNAs, and the corresponding mRNAs associated with each functional pathway were showed. **(E)** Key miRNAs regulatory network.

The 185 targeted mRNAs were analyzed using the DAVID functional annotation tool, which elucidates the biological significance of large gene lists. The BP terms of these genes were mainly enriched in oxidation-reduction process, cell migration process and especially metabolic process, including oxidation-reduction process, cholesterol biosynthetic process, steroid biosynthetic, steroid metabolic process, cholesterol metabolic process, lipid metabolic process, sterol biosynthetic, sterol metabolic process, and monounsaturated fatty acid biosynthetic process. The CC terms indicated enrichment for membrane-related functions, likely due to the receptor roles of some genes. The MF terms of the 185 targeted mRNAs included oxidoreductase activity, palmitoyl-CoA 9-desaturase activity, very-low-density lipoprotein particle receptor activity and hydrolase activity, supporting their involvement in metabolic process (Figures 3A–C). Importantly, five canonical pathways and their associated genes were annotated by KEGG pathway analysis. The result showed that the metabolic pathway encompasses genes related to steroid biosynthesis (*SQLE, LSS, HSD17B7, DHCR24*), arginine/proline metabolism (*ALDH18A1, PRODH2, AZIN2*), other amino acid metabolism (*GLUD1, MTHFD1L*), and glucose metabolism (*B4GALT1, GCNT3, GANC, XYLB*). Additionally, the vitamin digestion and absorption pathway (*LRAT, SCARB1, SLC19A3*) was also annotated by KEGG (Figure 3D). Subsequently, the genes annotated by the KEGG were linked to their regulatory miRNAs, suggesting that miR-497a-5p and miR-195a-5p mainly participated in steroid biosynthesis and glucose metabolism, whereas miR-326-3p and miR-122-5p participate in the amino acid metabolism. The remaining miRNAs were also found involved in the additional metabolic processes (Figure 3E). Notably, miR-122-5p plays a crucial role in the metabolic pathway, especially in arginine metabolism, with its target genes being PRODH2 and ALDH18A1.

### MiR-122-5p affect arginine metabolism by regulating PRODH2 and ALDH18A1

**FIGURE 4:**
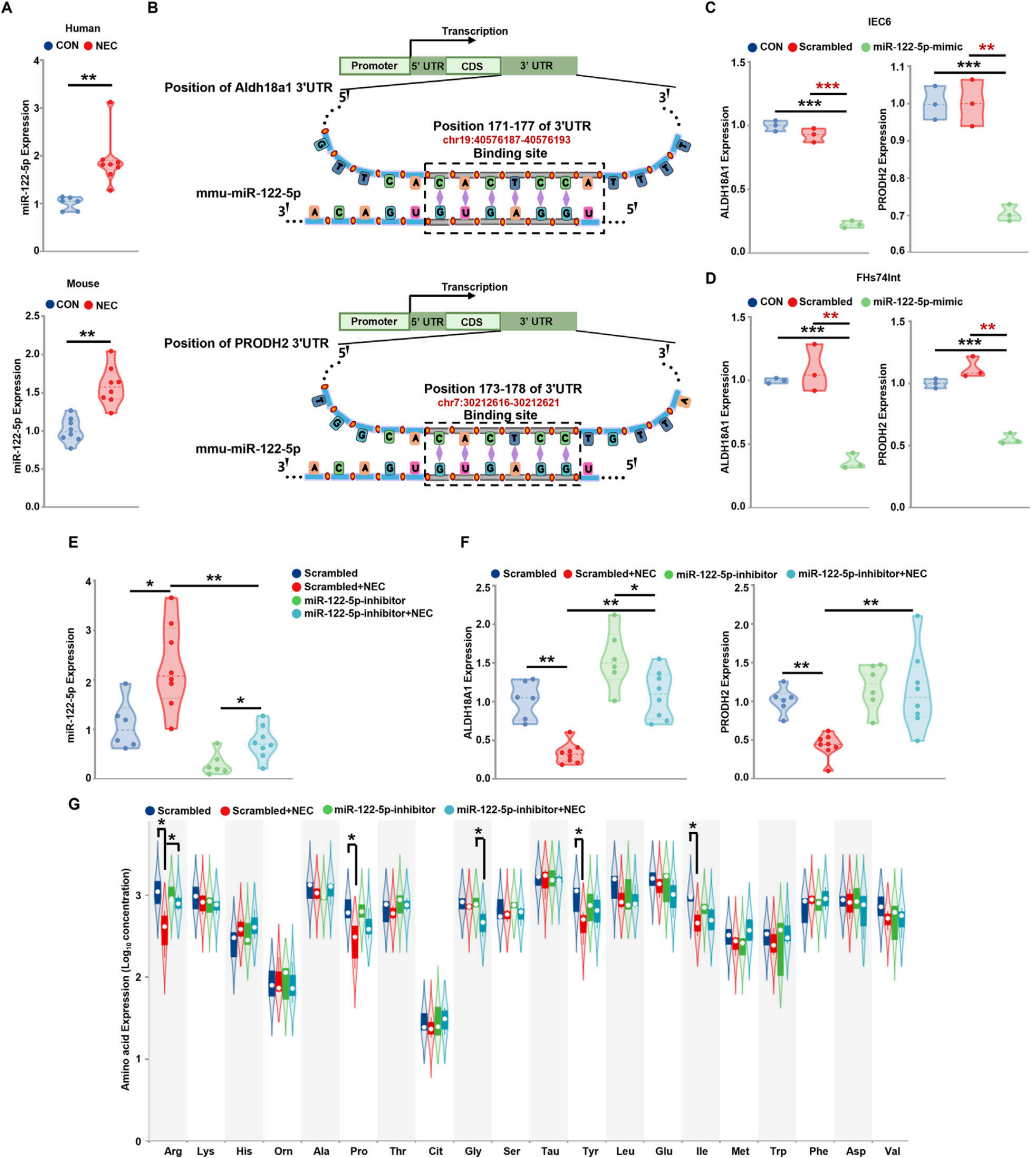
MiR-122-5p regulate NEC via PRODH2 and ALDH18A1 **(A)** The expression level of miRNA-122-5p in the human tissues and mice tissues. **(B)** Schematic diagram illustrated the binding site of miRNA-122-5p to PRODH2 and ALDH18A1 mRNAs. **(C, D)** PRODH2 and ALDH18A1 mRNAs expression level after miR-122-5p mimic sequence transfected IEC-6 cells and FHs74Int cells. **(E, F)** The statistics of miR-122-5p, PRODH2 and ALDH18A1 mRNAs levels in the scrambled group, miRNA-122-5p-inhibitor + scrambled group, NEC group, miRNA-122-5p-inhibitor + NEC group. **(G)** Analysis of intestinal amino acid concentrations (ug/g) in mice in the scrambled group, miRNA-122-5p-inhibitor + scrambled group, NEC group, miRNA-122-inhibitor + NEC group.

Then, we validate the expression level of miR-122-5p in NEC, compared to the control group, the miR-122-5p expression was significantly increased in the NEC group in the human tissues and mice tissues (Figure 4A). To further characterize the genes potentially regulated by miR-122-5p and their roles in NEC, miR-122-5p target prediction was performed. This analysis identified PRODH2 and ALDH18A1 as potential target genes of miR-122-5p. The binding of seed region on miRNA-122-5p to the target 3′UTR sequence of PRODH2 and ALDH18A1 mRNA were illustrated (Figure 4B). *In vitro*, miR-122-5p was overexpressed by transducing miR-122-5p mimic into FHs74Int and IEC-6 cells which significantly decreased the mRNA expression levels of PRODH2 and ALDH18A1 in NEC (Figures 4C, D). *In vivo*, compared to the scrambled group, the miR-122-5p expression was significantly increased in the NEC group. However, miR-122-5p expression was downregulated by transfection of miR-122-5p inhibitor (Figure 4E). Subsequently, we investigated whether the miR-122-5p downregulation influenced the expression of PRODH2 and ALDH18A1 and the results revealed that they increased following transfection with the miR-122-5p inhibitor (Figure 4F). We measured the concentrations of 20 amino acids in intestinal tissues obtained from the NEC mice and their scrambled groups. The results showed that the concentrations of arginine, proline, tyrosine, isoleucine were significantly lower in the mice with NEC compared to the scrambled group. Notably, the arginine concentration was restored when transfected with miR-122-5p inhibitor (Figure 4G). These findings showed that miR-122-5p may regulate arginine metabolism by targeting PRODH2 and ALDH18A1.

### MiR-122-5p inhibition alleviates intestinal injury by recovering intestinal stem cells and promoting intestinal regeneration in experimental NEC

**FIGURE 5:**
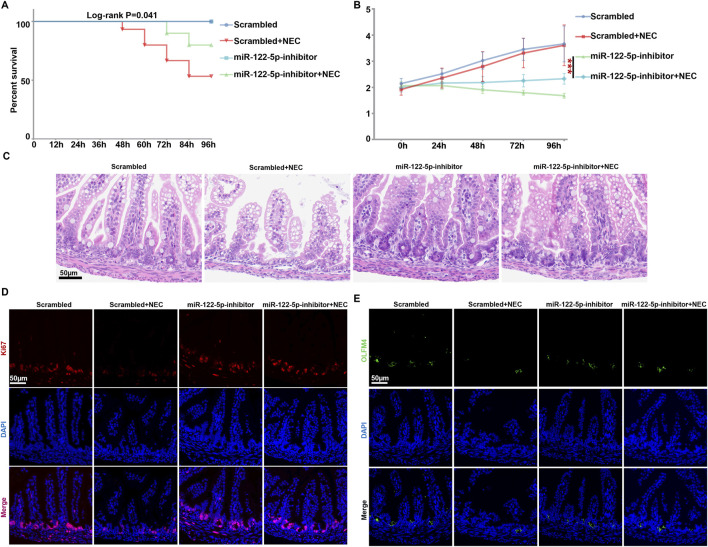
Intestinal proliferation and regeneration were rehabilitated with miR-122-5p inhibitor **(A)** Kaplan-Meier survival analysis of scrambled and NEC mice after transduction with miR-122-5p inhibitor. **(B)** Body weight of scrambled and NEC mice from each experimental group. **(C)** HE representative pictures of intestinal tissue from each experimental group. **(D–E)** Immunofluorescence representative micrographs of terminal ileum sections stained for Ki67 and Olfm4 from each experimental group.

Given that arginine can promote the healing of intestinal barrier through the self-renewal of ISCs (Hou QH. et al., 2020), we randomly allocated neonatal mice to either the scrambled group or NEC group, and then injected the mice with miR-122-5p inhibitor in both groups separately. The overall survival rate of the NEC group was significantly lower than that of the scrambled group but the miR-122-5p inhibition markedly improved the survival rate in NEC group (Figure 5A). The body weight of the scrambled group increased more rapidly with a better nutritional status, while the NEC group showed stagnation or decline in body weight over 96h. When inoculating the miR-122-5p inhibitor, there is a tendency to gain weight comparing to the NEC group (Figure 5B). In addition, typical NEC-like injuries, such as expansion, gas accumulation and even intestinal hemorrhage, were observed in the NEC group. These injuries were alleviated by the miR-122-5p inhibitor (Figure 5C). The proliferation of ISCs maintains the integrity of intestinal epithelium in NEC. In our study, the influence of miR-122-5p was assessed by immunofluorescence in the intestinal tissues of the experimental mice. We visualized the epithelial proliferation (Ki67) and ISCs activity (Olfm4) by immunofluorescence to evaluate the intestinal regeneration (Figures 5D, E). Both the epithelial proliferation and ISCs activity were decreased at the bottom of crypts in NEC mice compared to the scrambled group, but these effects were rescued by miR-122-5p inhibitor.

## Discussion

NEC is a devastating gastrointestinal disease especially to premature infants with the risk of long-term complications, such as intestinal stenosis, short bowel syndrome, parenteral nutrition-associated cholestasis, and neurodevelopmental disorders (Bazacliu and Neu, 2019; Hosokawa et al., 2023). To date, lines of evidence have shown that certain miRNAs have already been confirmed to be effective biomarkers for NEC, and target genes of these miRNAs maybe candidate treatment sites for NEC (Wu et al., 2021; Zhu et al., 2021). MiR-122-5p has been investigated in various kinds of diseases, such as lung cancer, pulmonary arterial hypertension sepsis and myocardial injury (Zheng et al., 2023; Wan et al., 2021; Zhang et al., 2023). A previous study revealed that miR-122-5p can regulate cancer growth by targeting p53, hindered cell proliferation and migration, and promoted apoptosis, thus providing feasible molecular target for the developing targeted drugs (Zheng et al., 2023). Additionally, it has been reported that miR-122-5p plays a crucial function to attenuate neuropathic pain by inhibiting pyruvate dehydrogenase kinase 4 (PDK4) expression (Wan et al., 2021). In this study, we utilized the miRNA high-throughput sequencing and subsequent bioinformatics analysis to investigate the potential pathogenic miRNA network and crucial genes in NEC. Preliminarily, we found 604 miRNAs by miRNA high-throughput sequencing, among which15 DEmiRNAs were detected, including five downregulated miRNAs and 10 upregulated miRNAs in NEC samples. Subsequently, the DemiRNA-mRNA regulatory network was constructed. The enrichment analysis demonstrated that miR-122-5p plays an important part in the pathogenesis of NEC by targeting the transcription factors ALDH18A1 and PRODH2, and the DEmRNAs were significantly enriched in metabolism pathway, including arginine metabolism.

Arginine is an amino acid that acts as a precursor for proteins and nitric oxide synthase, can be synthesized in small intestine (Shah et al., 2017). It has been revealed that low expression level of circulating arginine is associated with the occurrence of NEC. The inhibition of histone deacetylase 8 (HDAC8) expression facilitates arginine synthesis via acetylation of histone three lysine9 (acetyl-H3K9) regulation thus protecting mice from NEC (Guo et al., 2023). Additionally, dietary supplementation of L-arginine has been confirmed to reduce the severity of intestinal injury by promoting nitric oxide synthesis in an experimental model of hypoxemia/reoxygenation-induced NEC (Akisu et al., 2002). Premature infants have low arginine and intake, Kam and colleagues discovered that the expression of arginine synthesizing enzymes, including ALDH18A1, ASL, ASS1, CPS1, GLS, OAT, OTC and PRODH were significantly decreased in the in NEC tissues (Leung et al., 2016). This finding is consistent with our result that arginine concentration was significantly decreased in the NEC group. In this study, miR-122-5p inhibitor showed a protective role in NEC mice, increasing the arginine concentration after transfection of the miR-122-5p inhibitor. Furthermore, our RT-qPCR results showed that transfection of miR-122-5p inhibitor can lead to the increasing expression of PRODH2 and ALDH18A1 both in the IEC-6 cells and the mice samples. These outcomes revealed that miR-122-5p downregulation can maintain the arginine metabolism via increasing the expression level of PRODH2 and ALDH18A1, thus exerting a valid protective role to NEC.

Although the pathogenesis of NEC remains incompletely understood, an acquired loss of ISCs may provide a partial influence, resulting in a gross inability to recover intestinal mucosa (Di et al., 2022; Kim et al., 2022). The ISCs were localized within the intestinal crypts and play a pivotal role for damage-induced intestinal regeneration to maintain the integrity and viability of the epithelial layer (Snippert et al., 2010; Huan et al., 2017). Previous study has demonstrated that intestinal injury leads to expansion of ISCs and facilitates cell proliferation, thereby preventing further damage to intestine (Liu et al., 2023). It has been demonstrated that activation of Toll-like-receptor 4 (TLR4) on ISCs induced endoplasmic reticulum (ER) stress, leading to ISCs apoptosis and NEC (Afrazi et al., 2014). Additionally, the Wnt/β-catenin signaling pathway, BMP, growth factors, and Notch cascades were proved to be essential for the proliferation and maintenance of ISCs (Schuijers and Clevers, 2012; Li et al., 2019). As for the influence of L-arginine on ISCs, mice and small intestinal (SI) organoid models were conducted, revealed that L-arginine supplementation stimulated Wnt2b secretion in CD90^+^ stromal cells through the mammalian target of rapamycin complex 1 (mTORC1) signaling pathway, thus induce the expansion of the ISC (Hou Q. et al., 2020). In the present study, compared with the control group, the proliferation and ISCs activity were decreased in the NEC group, but were rescued by miR-122-5p inhibitor treatment.

In summary, we have identified miR-122-5p was highly expressed in the NEC, while miR-122-5p inhibitor can act as a protective factor in NEC development via regulating arginine metabolism. The interactions of miR-122-5p, PRODH2, ALDH18A1and arginine provide a new potential target for diagnostic and therapeutic option for NEC.

However, there still exists some limitations. For instance, it is still urgently needed to reveal the signaling pathways that might be involved in the regulatory mechanism of miR-122-5p. Further exploration is needed in the future.

## Data Availability

The original contributions presented in the study are publicly available. This data can be found here: https://www.ncbi.nlm.nih.gov/ GSE212694, GSE286479.
